# Role of patient safety attitudes between career identity and turnover intentions of new nurses in China: A cross-sectional study

**DOI:** 10.3389/fpubh.2022.981597

**Published:** 2022-11-02

**Authors:** Man Zhang, Xutong Zheng, Changchang Chen, Jiaxin Fang, Huan Liu, Xiancui Zhang, Hongjuan Lang

**Affiliations:** ^1^School of Nursing, Yan'an University, Yan'an, China; ^2^School of Nursing, Fujian University of Traditional Chinese Medicine, Fuzhou, China; ^3^Department of Nursing, Air Fourth Military Medical University, Xi'an, China; ^4^Department of Nursing, Beijing University of Chinese Medicine, Beijing, China; ^5^Department of Hemodialysis, The First Affiliated Hospital of Wan'nan Medical College, Wuhu, China; ^6^Medical Examination Center, The First Affiliated Hospital of Wan'nan Medical College, Wuhu, China

**Keywords:** patient safety, new nurses, turnover intention, professional identity, safety culture

## Abstract

**Background:**

Patient safety is a key priority for healthcare systems, which is not only about the safety and quality development of health care but also about the safety of patients' lives. However, there has been little research exploring the relationship between new nurses' willingness to leave, patient safety culture, and professional identity. This study was to explore patient safety for new nurses, examine the relationship between professional identity, patient safety culture, and turnover intentions of newly recruited nurses in China, and validate the mediating role of patient safety culture.

**Methods:**

From August 2019 to September 2021, we collected data from newly recruited nurses in 5 large tertiary public hospitals in Anhui Province, China using a questionnaire survey. Descriptive analysis, a univariate analysis, Pearson correlation analysis, and mediated regression analysis were used to estimate the current status of patient safety attitudes and the effect of safety culture on career identity and turnover intentions among newly recruited nurses.

**Results:**

The turnover intention of 816 newly recruited nurses was 14.16 ± 3.14%. Patient safety culture was positively associated with career identity (*r* = 0.516, *P* < 0.01) and negatively associated with turnover intentions (*r* = −0.437, *P* < 0.01), while patient safety was also a partial mediator between career identity and turnover intentions.

**Conclusions:**

The results showed that the low patient safety attitudes of new nurses in China should not be ignored. The impact of professional identity on patient safety has important practical implications for promoting a culture of safety among new nurses and reducing turnover rates.

## Introduction

The event of patient safety is considered any process, act of omission, or commission, thus resulting in hazardous healthcare conditions and/or unintended harm to the patients (1). Reporting patient safety incidents is an effective way to improve patient safety. At the core of any health care service is patient safety and quality of care, which has become a challenge for health systems (2). Patient safety culture included group cooperation within the unit, organizational learning, patient safety management support, overall patient safety awareness, error feedback and communication, and openness of communication (3, 4). Studies investigating the cognition of patient safety culture in adult intensive care unit (ICU) professional teams found the team's safety attitude weak (5). A lack of safety culture can cause adverse effects and serious harm to the patients (6). Developing a culture of safety among new nurses is an essential part of efforts to improve patient safety and quality of care in the nursing environment.

In healthcare systems, the most common occupation for identifying, intercepting, and correcting life-threatening errors is nursing (7). The current study found that the turnover intention of unused nurses is affected by both internal and external components, such as person components counting gender, self-efficacy, work-family conflict, work environment, working hours, and so on (8). Currently, the turnover of nurses has been a major factor in nursing shortages, which hinders the development of nursing research and the sustainable development of practice (9). One of them is the shortage of new registered nurses, with studies showing that more than 10% of nurses leaving each year were newly registered nurses, 29% of the unused nurses changed work frequently after 12 months, 33–62% of medical attendants will be exchanging or taking off within 5 years (10).

While many efforts have been paid off, solving the problem is far from satisfactory. Previous systematic reviews have shown that nurses working in overloaded and highly demanding environments often suffer from anxiety and burnout (11). It is also found that a safety attitude has a negative relation with turnover intention (12). A responsible safety attitude can lead to fewer medical errors, thus causing better response and appraisal from patients and nursing supervisors (13). The positive feedback may enhance the confidence and willingness to work with new nurses, consequently leading to less turnover. However, there is a huge contradiction between the nurses' inner expectations and reality. Highly educated nurses, in particular, face contradictions in their clinical work and struggle to gain professional approval (10). Studies have shown that nurses are less satisfied there if they have a lower level of job identification after graduation. The emotional stress is gradually reduced by increasing the job satisfaction of nurses, which positively affects their willingness to leave (14). A sense of accomplishment and job satisfaction are essential factors in reducing the propensity of new nurses to leave (15).

**Figure 1:**
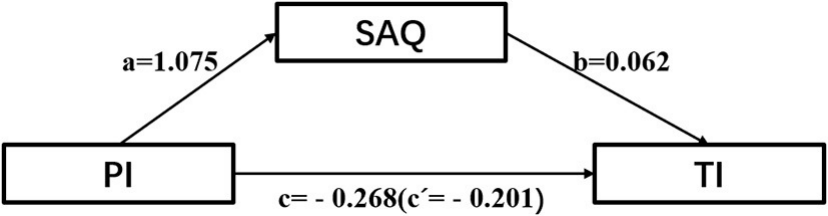
The mediating role of safety attitude.

The questionnaires can be used to investigate clinicians and staff about aspects of their team, work area, or hospitals, such as communication about safety hazards, transparency, teamwork, and leadership (16). The relationship between a new nurse‘s safety culture and professional identity and willingness to leave is unclear. If there would be some relationship between the physician's patient safety culture and professional identity is still unknown. Yet, the type of relationship that exists between new nurses‘ safety culture and professional identity and turnover intention remain unclear. Thus, this study aims to explore the relationship between new nurses' patient safety, professional identity, and resignation intention of new nurses and help to determine the relationship among three factors by using questionnaires. Based on the literature review above, we developed a model to test the following hypotheses (Figure 1): (a) safety attitude acts as the mediating role between occupational identity and turnover intention; (b) professional identity directly influences safety attitude; (c) safety attitude directly influences turnover intention; (d) professional identity directly affects turnover intention; (e) professional identity indirectly influences turnover intention through safety attitude.

## Methods

### Study design and participants

A questionnaire survey was conducted from August 2019 to September 2021 on 816 new nurses in 5 tertiary hospitals in Anhui Province, China. Finally, 816 questionnaires were collected (all are valid). The selected 5 hospital levels, department settings, and medical levels were all similar. Before the investigation, we first communicated, coordinated with the nursing management of the hospitals, and explained the purpose of the investigation to section matrons for cooperation to obtain consent. Nurse managers were asked to distribute electronic questionnaires to new nurses on the ward, using a uniform guideline and stating the completion deadline. Nurses were asked to complete the questionnaire in their leisure time to avoid errors and missing items. Inclusion criteria of participants for this study: (1) 1–3 years of graduation; (2) having a nursing license and currently practicing in a clinical setting; (3) being informed consent and voluntary participation in this study. Exclusion Criteria: (1) Not a first-time employee; (2) Nurse Internship. A questionnaire will be considered invalid if it complies with one of the circumstances below: (1) More than 10 items of questionnaires were filled with the same answer continuously; (2) The time took to fill out the questionnaire was too long or too short; (3) Any item on the questionnaires was omitted.

### Instrument

#### Demographic characteristics

Demographic data of new nurses were collected, including gender, age, marital status, time of entry, daily working hours, personal interests, and nursing inclinations, and were collected.

#### Occupational identity scale

The Career Identity Scale includes occupational cognition assessment, occupational social support, occupational social skills, occupational self-reflection, and occupational frustration (17). A 5-point Likert scale (1 = strongly disagree, 2 = disagree, 3 = fair, 4 = agree, 5 = strongly agree) with 30 entries was used, with higher scores indicating higher levels of professional identity for nurses.

#### The safety attitude questionnaire scale

The Safety Attitude Questionnaire (SAQ) scale version of Chinese mandarin translated by Guo et al. is used in the research (18). In the SAQ scale, a total of 31 items were divided into 6 dimensions consisting of teamwork, safety climate, perceptions of management, job satisfaction, working conditions, and stress recognition to measure nurses' attitudes toward patient safety. Each item had a 5-point scale (1 = strongly disagree to, 3 = Neutral to, 5 = strongly agree). The higher the total score was, the more safety-conscious the new nurses were.

#### Turnover intention scale

The study used the Intention to Leave Scale developed by Michaels to measure the intention to leave of new nurses to leave (19). The number of items with 3 dimensions was 6. Among them, the 1st and 6th entry constituted the turnover intention I; 2nd and 3rd entry constituted the turnover intention II; 4th and 5th entry constituted the turnover intention III. The turnover intention I, II, and III indicated the probability of employees quitting their current job, the motivation of employees to find other employment, and the likelihood of employees obtaining outside jobs, respectively. The scale was rated on a 4-point Likert scale (1 = never, 2 = rarely, 3 = occasionally, 4 = frequently). A total mean score of ≤ 1 indicated a very low turnover intention; >1 and ≤ 2 indicated a low turnover intention; >2 and ≤ 3 indicated a high turnover intention, and>3 indicated a very high turnover intention.

### Data analysis

This study used IBM Statistical Product Service Solutions (SPSS) version 26.0 to perform statistical analysis. The general profile characteristics of the participants were compared by independent samples *t*-test or one-way analysis of variance (ANOVA). First, Pearson correlation evaluation and covariance diagnosis were conducted to assess patient safety attitudes, turnover intentions, and willingness to leave. The PROCESS macro (version 3.3 by Andrew F. Hayes) for SPSS was conducted to test for mediating effects and LS with 5,000 bootstrap samples, with patient safety as the mediating variable, intention to leave as the dependent variable, and career identity as the independent variable to calculate direct effects (path c‘), indirect effects (path a^*^b) and total effects (path c). Mediating effect was significant when the 95% confidence interval (CI) of the indirect effect showed no *P*-value < 0.05.

## Results

### Demographic characteristics of new nurses

A total of 816 respondents meeting the criteria for inclusion were included in this study. Table 1 presented the general demographic characteristics of the respondents, including gender, age, marital status, time of entry, daily working hours, personal interests, and inclinations of nursing. Among all respondents, 87.3% were female and 74.2% had a bachelor's degree or higher. On average, 66.5% of new nurses worked 8–10 h per day, and 16.4% had thoughts of quitting recently. The hospitals selected for this study had some level of medical, teaching, and scientific research capabilities.

**Table 1:** General information of new nurses (*n* = 816).

**Variables**	***n***	**Percentage (%)**
**Gender**		
Male	104	12.7
Female	712	87.3
**Age**		
21–23	346	42.4
24–26	407	49.9
27–28	57	7.0
29	6	0.7
**Education**		
Junior college	1	0.1
College	210	25.7
Undergraduate	579	71.0
Master and above	26	3.2
**Marital status**		
Unmarried	753	92.3
Married	62	7.6
Other	1	0.1
**Children's status**		
Sterile	781	95.7
Fertile	25	3.1
Current	10	1.2
**Pregnancy year of entry**		
2017	210	25.7
2018	323	39.6
2019	283	34.7
**Average monthly revenue**		
≤ 2,000	160	19.6
2,001–4,000	248	30.4
4,001–6,000	224	27.5
6,001–8,000	138	27.5
≥8,000	46	5.6
**Preference**		
Related to the nursing profession	106	13.0
Not relevant	544	66.7
General related	544	20.3
**Average daily working time (h)**		
≤ 8	242	29.7
8–10	242	66.5
10–12	28	3.4
≥12	3	0.4
**Average number of hours of patient contact per day (h)**		
0–2	24	2.9
2–4	73	8.9
4–6	235	28.8
6–8	484	59.3
**Recent resignation thoughts**		
Yes	134	16.4
No	682	83.6
**Self-assessment of physical condition**		
Very poor	9	1.1
Poor	66	8.1
Fair	491	60.2
Better	217	26.6
Very good	33	4.0
**Number of nursing adverse events**	645	79.0
1	100	12.3
2	37	4.5
3	26	3.2
4	4	0.5
5	2	0.2
10	2	0.2

### Correlations between study variables

In Table 2, correlations between study variables including professional identity (PI), safety attitude questionnaire (SAQ), and turnover intention (TI) were presented. On average, the scores of attitude toward safety, turnover intention, and career identity were 114.54, 14.16, and 39.65, respectively. Turnover intention appeared a negative relationship to security state of mind (*r* = −0.437, *P* < 0.01), and occupational identity (*r* = −0.502, *P* < 0.01). Furthermore, a positive relationship between professional character and attitude toward patient safety was observed (*r* = 0.516, *P* < 0.01). These results show that there is a significant correlation between the variables.

**Table 2:** The correlation analysis among research variables.

**Variables**	**Mean ±SD**	**SAQ**	**TI**	**PI**
SAQ	114.54 ± 13.296	1	–	–
TI	14.16 ± 3.14	−0.437^**^	1	–
PI	39.65 ± 6.282	0.516^**^	−0.502^**^	1

PI, professional identity; SAQ, safety attitude questionnaire; TI, turnover intention;

** P < 0.01.

### The difference between characteristics of new nurses and scores of multiple variables

The difference between the characteristics of new nurses and scores of multiple variables was also compared, as shown in Table 3. The scores of various variables (PI, SAQ, TI and et al.) were related to occupational identity, including some characteristics of respondents, such as age, gender, education background, entry time, and monthly income of new nurses.

**Table 3:** Difference analysis among variables.

**Characteristics**	**PI = Mter**	**SAQ = Meri**	**TI = Mean ±SD**
**Gender**			
Male	38.25 ± 7.017	116.31 ± 14.372	4.31 ± 1.462
Female	39.85 ± 6.264	114.28 ± 13.122	4.38 ± 1.512
F/t	−2.206	1.455	−0.488
**Age**			
21–23	39.60 ± 6.320	115.14 ± 14.009	4.41 ± 1.498
24–26	39.65 ± 6.506	114.01 ± 12.594	4.35 ± 1.535
27–28	39.61 ± 6.123	113.91 ± 13.908	4.44 ± 1.337
29	43.17 ± 3.488	121.33 ± 10.967	3.50 ± 1.378
F/t	0.615	1.021	0.833
**Education**			
Junior college	30.00	92.00 ± 13.656	6.00
College	41.26 ± 5.964	114.58 ± 13.140	4.27 ± 1.618
Undergraduate	39.08 ± 6.410	114.64 ± 13.140	4.41 ± 1.472
Master and above	39.65 ± 6.893	112.81 ± 13.720	4.35 ± 1.263
F/t	6.907^**^	1.117	0.876
**Marital status**			
Unmarried	39.75 ± 6.403	114.70 ± 13.260	4.37 ± 1.512
Married	38.42 ± 6.091	112.50 ± 13.773	4.45 ± 1.338
Other	37.00	115.00	4.00 ± 1.265
F/t	1.340	0.787	0.117
**Children's status**			
Sterile	39.65 ± 6.415	114.48 ± 13.282	4.39 ± 1.512
Fertile	40.56 ± 5.181	117.52 ± 13.125	3.96 ± 1.338
Current pregnancy	37.60 ± 6.653	111.60 ± 15.005	4.40 ± 1.265
F/t	0.770	0.880	0.981
**Year of entry**			
2017	40.04 ± 6.570	114.94 ± 13.207	4.34 ± 1.552
2018	39.82 ± 6.407	113.21 ± 13.660	4.37 ± 1.478
2019	39.17 ± 6.204	115.75 ± 12.844	4.41 ± 1.504
F/t	1.323	2.899	0.112
**Average monthly revenue**			
≤ Even	39.09 ± 6.382	115.09 ± 13.838	4.54 ± 1.570
2,001–4,000	39.34 ± 6.166	113.49 ± 13.686	4.48 ± 1.478
4,001–6,000	40.21 ± 6.449	115.39 ± 12.467	4.17 ± 1.572
6,001–8,000	40.34 ± 6.357	115.00 ± 13.262	4.32 ± 1.362
≥600	38.46 ± 7.064	112.70 ± 13.285	4.43 ± 1.424
F/t	1.694	0.950	1.945
**Preference**			
Related to the nursing profession	33.98 ± 6.597	106.27 ± 12.544	5.13 ± 1.339
Not relevant	39.14 ± 5.688	113.40 ± 11.609	4.52 ± 1.398
General related	44.94 ± 4.169	123.55 ± 14.131	3.42 ± 1.498
F/t	133.183^**^	71.005^**^	56.450^**^
**Average daily working time (h)**			
≤ 8	40.86 ± 5.632	116.88 ± 12.147	4.22 ± 1.535
8–10	39.28 ± 6.611	113.97 ± 13.521	4.39 ± 1.488
10–12	36.50 ± 6.333	106.29 ± 14.483	5.29 ± 1.301
≥14	38.00 ± 6.000	105.67 ± 8.083	5.00 ± 1.000
F/t	5.919^**^	7.019^**^	4.494^**^
**Average number of hours of patient contact per day (h)**			
0–2	42.42 ± 4.690	116.83 ± 13.541	4.04 ± 1.459
2–4	39.51 ± 6.681	114.99 ± 11.687	3.99 ± 1.307
4–6	39.89 ± 6.178	115.27 ± 12.308	4.38 ± 1.476
6–8	39.42 ± 6.487	114.00 ± 13.963	4.45 ± 1.541
F/t	1.838	0.771	2.393
**Recent resignation thoughts**			
Yes	34.97 ± 6.848	105.50 ± 14.232	5.53 ± 1.237
No	40.57 ± 5.868	116.31 ± 12.361	4.15 ± 1.448
F/t	−8.847^**^	−8.207^**^	10.329^**^
**Self-assessment of physical condition**			
Very poor	33.67 ± 9.327	94.11 ± 9.892	5.89 ± 1.269
Poor	36.94 ± 6.663	107.97 ± 14.536	5.00 ± 1.392
Fair	39.40 ± 6.202	113.64 ± 12.618	4.45 ± 1.456
Better	40.76 ± 6.049	117.98 ± 12.055	4.03 ± 1.507
Very good	43.06 ± 6.557	123.88 ± 15.518	3.88 ± 1.709
F/t	9.516^**^	19.174^**^	9.507^**^
**Number of nursing adverse events**	39.86 ± 6.396	115.40 ± 13.256	4.29 ± 1.506
1	39.16 ± 5.872	111.11 ± 12.131	4.56 ± 1.409
2	37.68 ± 6.864	111.97 ± 14.614	4.81 ± 1.506
3	39.65 ± 6.431	112.19 ± 13.145	4.96 ± 1.280
4	39.25 ± 8.655	105.00 ± 20.248	4.75 ± 2.217
5	29.00 ± 1.414	99.00 ± 8.485	7.00 ± 0.00
10	45.00 ± 1.414	120.50 ± 9.192	4.50 ± 3.536
F/t	1.981	2.825^**^	2.895^**^

PI, professional identity; SAQ, safety attitude questionnaire; TI, turnover intention;

** P < 0.01.

### Turnover intention scale sub-dimension score

Scores of each dimension of turnover intention were indicated in Table 4. The scores of nurses' TI I, II, and III were 4.31, 4.38, and 5.47, respectively. The nurses' turnover intention III scores were higher than those of the other two dimensions.

**Table 4:** Scores of each dimension of turnover intention.

**Items**	**Scores ($\bar{\text{x}}$ ±s)**	**Means ($\bar{\text{x}}$ ±s)**	**The metric value (%)**
TI-I	4.31 ± 1.458	2.157 ± 0.7288	53.9
TI-II	4.38 ± 1.505	2.188 ± 0.7523	54.8
TI-III	5.47 ± 1.127	2.753 ± 0.5635	68.4
Aggregate score	14.16 ± 3.410	2.359 ± 0.568	59.0

### Mediation regression models of study variables

To illustrate the relationship between the variables more clearly, mediation regression models of study variables were shown in Table 5. It was interpreted as follows: the direct effect of occupational identity on patient safety was 1.075 (*P* < 0.01) and positive; patient safety attitude negatively affected the turnover intention with an effect of −0.062 (*P* < 0.01); Meanwhile, the effect of professional identity on turnover intention was −0.201 (*P* < 0.01). Therefore, a^*^b^*^c was positive (Figure 1). It was indicated that patient safety attitude, as a mediating variable, can explain the relationship between new nurses' career identity and willingness to leave.

**Table 5:** Results of mediation analyses.

**Paths**	**Direct effects**	**Indirect effects**	**Total effects**	**95%CI**
PI—SAQ	1.075^**^	–	1.075^**^	(0.952, 1.198)
SAQ–TI	−0.062^**^	–	−0.062^**^	(−0.08, −0.045)
PI–TI	−0.201^**^	−0.067^**^	−0.268^**^	(−0.3, −0.236)

N = 816; PI, professional identity; SAQ, Safety attitude questionnaire; TI, turnover intention;

** P < 0.01.

## Discussion

Turnover intention is defined as the possibility that employees will leave a job within a certain period (20). It is considered to be one of the best factors to predict turnover behavior (21). In this study, the mean intention to turnover of the 816 newly recruited nurses was 14.16 ± 3.14, which was significantly higher than the turnover intention of nurses in other places in China (22–25). The above situation may be related to the selected area in this study, Anhui Province, where the emergency department workload of nurses is more intensive. In addition, new nurses are likely to have a strong desire to leave since they are unable to adapt to a professional and intensive working environment (26). Nurses who work an average of 10 h a day or more a day are 2.5 times more likely to leave than those who work shorter shifts (27). Nurses who retain their posts and meet the annual assessment requirements are recommended to be awarded honorary certificates or medals, to have their salaries adjusted or adequately subsidized, to enhance the sense of identity and honor of new nurses in their jobs, and to reduce turnover rate. Patient safety attitudes were higher among nurses in regions like Europe and America, inwards like ICUs, and nurses with advanced degrees like graduate nursing students (28). In our study, scores of new nurses' patient safety attitudes were 114.54 ± 13.296, significantly lower than members of a multidisciplinary team in a medium-sized hospital in other countries (29–31). The average score of all dimensions was in the range of 10–30, and our results may to some extent explain the lower patient safety attitudes of new nurses, considering differences in regions, departments, and tasks. Regarding the current situation of the lower patient safety attitude of new nurses in Anhui, China, it is suggested to focus on each dimension of patient safety attitude from high to low recognition, working condition, job satisfaction, management perceptions, teamwork climate, and safety climate (32). Our study showed that factors such as whether one's hobbies corresponded to one's nursing specialty, average daily working hours, recent thoughts about quitting, self-rated physical condition, and the number of adverse events each influenced the patient safety attitudes of new nurses to different extents. Hence, it is more important to pay more attention to various factors affecting patient safety attitudes and take effective and appropriate measures to improve the negative factors. Nurse managers should organize group activities to keep the nursing team in good physical condition, reasonably allocate the number of nursing staff in each position, and reduce the average working time to avoid adverse events. Promoting legal knowledge among new nurses enhances the professional identity and sense of honor of new nurses, such as the Chinese version of the Nurses Regulation, to learn how to use the law to protect our nurses' legitimate rights.

The professional identity of nurses is affected by different inside and outside components. Based on pre-service preparation, the face-to-face coaching of senior nursing staff can help to enhance their working competence and build a professional team of nursing (33). In our study, the professional identity of new nurses was found to be poor. In this regard, the competent personality of modern medical staff was lower than that of nurses in Beijing (34), which may be related to the impact of external components such as the improvement of regional financial, social, and educational levels. As for new nurses in Anhui province, China, it was found that the level of instruction, whether my side interests are related to the nursing calling, normal working hours, later considerations of cessation, and self-assessed physical condition were vital components affecting unused nurses' proficient character. Our results showed that the new nurses' professional identity was higher than that of nursing students (35), which may be related to the truth that clinical nurses have more clinical involvement than nursing students. Therefore, it is necessary to further explore the relationship between gender and the good personality of modern nurses in the context of China. The better the self-rated fitness of medical staff, the higher their safety culture scores they got. It is strongly advised that nursing managers should pay attention to the physical well-being of medical attendants, reasonable shift arrangements, and avoid the nurses' long hours of nursing work in a state of exhausted and intense nursing. In addition, nurse researchers should actively develop and explore single-patient care management and new care models to help new nurses improve their professional competence and thereby improve patient safety attitudes.

Understanding safe attitudes are crucial when new nurses have turnover thoughts at work (36, 37). Our study found that the patient safety attitudes of new nurses mediated the role of career identity on turnover intentions, which is consistent with our study hypothesis. Notably, there is a lack of other studies that give direct support to our hypothesis. Moreover, we found a significant direct positive effect of new nurses' professional identity on patient safety attitudes, which is similar to previous findings (38, 39). It may also be related to the fact that new nurses working in the profession are initially informed about safety-related content, which facilitates the development of professional identity in terms of patient safety attitudes. These new nurses can upgrade their understanding of security behaviors at work because these conscious of security can successfully decrease the readiness of new nurses to leave. Precisely due to the vital role that patient safety attitudes played in reducing new nurses and intentions to go. The management should consider promoting the magnetic hospital's establishment with regional characteristics, strengthening organizational culture, and reducing the willingness of new nurses to leave.

## Conclusions

Patient safety has long been considered a crucial global health issue. In our study, we found that new nurses' lack of patient safety knowledge and awareness was highly correlated with their sense of professional identity and turnover intention. Exploring training programs suitable for new nurses can not only reduce the nurses of attrition but also improve the ability of decision-makers to grasp new nurses comprehensively and better cultivate their clinical leadership.

## Data availability statement

The original contributions presented in the study are included in the article/supplementary material, further inquiries can be directed to the corresponding author/s.

## Ethics statement

Ethical review and approval was not required for the study on human participants in accordance with the local legislation and institutional requirements. Written informed consent from the participants' legal guardian/next of kin was not required to participate in this study in accordance with the national legislation and the institutional requirements.

## Author contributions

MZ and XZhe conducted calculations, analyzed results, and drafted the manuscript. MZ, CC, and JF were responsible for the overall design of the research, conducted, and designed the analysis framework. XZha and HLi joined in the data collection. XZhe, JF, and HLa revised the paper. All authors approved of the current version of this manuscript for publication.

## Conflict of interest

The authors declare that the research was conducted in the absence of any commercial or financial relationships that could be construed as a potential conflict of interest.

## Publisher's note

All claims expressed in this article are solely those of the authors and do not necessarily represent those of their affiliated organizations, or those of the publisher, the editors and the reviewers. Any product that may be evaluated in this article, or claim that may be made by its manufacturer, is not guaranteed or endorsed by the publisher.
